# Assessing hand dysfunction in cervical spondylotic myelopathy

**DOI:** 10.1371/journal.pone.0223009

**Published:** 2019-10-28

**Authors:** Zachary A. Smith, Alexander J. Barry, Monica Paliwal, Benjamin S. Hopkins, Donald Cantrell, Yasin Dhaher

**Affiliations:** 1 Department of Neurological Surgery, Northwestern University, Chicago, Illinois, United States of America; 2 Shirley Ryan Ability Lab, Northwestern University, Chicago, Illinois, United States of America; 3 Department of Radiology, Northwestern University, Chicago, Illinois, United States of America; Universiteit Antwerpen, BELGIUM

## Abstract

Cervical Spondylotic Myelopathy (CSM) is a common spinal condition that presents as hyperreflexia, loss of dexterity, and strength. Despite its prevalence, little is known about the specific neuromechanical deficits that constitute overall disability in CSM. Compression on MRI doesn’t exclusively relate to disability. Moreover, clinical assessment often relies on the subjective exams and self-reported questionnaires. Therefore, the purpose of this study was to assess hyperreflexia, proprioception, and loss of strength, and its association with common MRI scales.

**Methods:**

Twenty patients with CSM and 17 controls were recruited. Clinical scores of modified Japanese Orthopedic Association (mJOA) and Nurick were collected. MRI based compression grades such as cord distortion were assessed. Hand dysfunction was tested using a custom motorized apparatus. Subject’s forearm was placed in a cast and positioned such that their metacarpophalangeal (MCP) joint was vertically aligned with the motor shaft. Surface electromyographic sensors were placed on flexor digitorum superficialis (FDS) and extensor digitorum communis muscles. Hyperreflexia was measured as the FDS muscle activation during reflex when the MCP joint was moved from flexion to extension at 300°/sec. Proprioception was quantified as the angle of detection in absence of visual or auditory cues (subjects were blindfolded and given noise-cancelling headphones). Strength was measured as the maximum isometric force at the MCP joint. 2-sample t-test (p<0.05) were performed to assess significant differences in reflexes, proprioception and strength among patients and controls (SPSS software version 24).

**Results:**

Patients reported higher Nurick (1.90±1.0 vs 0±0, p<0.001) and lower mJOA scores (14.3±1.9 vs 18.0±0, p<0.001) as compared to controls. Similarly, patients with CSM had increased reflexes (peak FDS EMG) (0.073±0.096 vs. 0.014±0.010, p = 0.019). Patient proprioception was significantly worse; mean angle of detection was greater than twice as high in patients (9.6± 9.43°) compared to controls (4.0± 2.3°), p = 0.022. MRI based compression ratio (CR) was a significant predictor of hyperreflexia, CR<0.44 resulted in steep increase in reflex activity. Fifteen of the 20 patients who completed follow up testing at 6 months after surgery show substantial clinical improvement in reflexes and proprioceptive angle, while repeated testing in controls were unchanged.

**Conclusion:**

In conclusion, hyperreflexia and decline in proprioception are the main drivers of hand disability in patients with CSM. Of multiple scales, only a select few MRI scales (such as compression ratio) were predictive of increased reflexes. The study describes a pre-clinical testing apparatus to quantitatively and objectively assess primary presenting symptoms in CSM. This pilot apparatus has the potential to evaluate treatment efficacy through repeated testing. Objective testing of hand dysfunction can help inform the design of clinically feasible devices, guide MRI biomarker analysis, and improve our understanding of the progression of neurological injury in this patient population.

## Introduction

Cervical spondylotic myelopathy (CSM), a condition characterized by progressive spondylosis of the cervical spine, commonly leads to poor dexterity and diminished strength in the hands [1–3]. Commonly, the cardinal presenting symptoms of CSM include a progressive loss of skilled fine finger movement and grip strength [4]. These complaints may be the result of a loss of strength, proprioceptive impairment, and the impact of abnormally enhanced stretch reflexes [4]. The modern spinal assessment, often conducted in an outpatient clinic, relies heavily upon subjective tests of motor and sensory impairment, clinical disability scales, and magnetic resonance (MR) imaging [5], [6]. There is little known about how specific isolated clinical deficits (strength, proprioception, and hyperreflexia) lead to a cumulative picture of clinical disability [7]. An improved understanding of what drives clinical disability in CSM patients may improve the clinician’s ability to make decisions on the timing of treatment and need for close follow-up when surgery is not offered, especially in patients with mild myelopathy. Furthermore, precise evaluations after surgery will inform discussions of prognosis and demonstrate the role of physical therapy and rehabilitation in recovery. The preceding discourse describes a method to determine function-specific and reproducible measures of neurological impairment in the CSM population.

In the present study, we seek to characterize hand dysfunction in patients with cervical myelopathy using objective measures of sensorimotor function. We investigate strength, proprioception, and hyperreflexia at the metacarpophalangeal joint (MCP), utilizing the flexor and extensor hand muscles in patients with cervical myelopathy and a cohort of healthy controls. We hypothesize that there will be a differential effect in these three neuromechanical parameters and patients will exhibit enhanced reflexes, lower strength, and proprioceptive loss as compared to controls. These may represent tract-specific ascending and descending fiber deficits that culminate in a patient’s more aggregate presentation of clinical disability. Cervical myelopathy patients can commonly present quite heterogeneously and there can often be clinical symptoms either exceeding or less than would be expected for the degree of compression. We believe that there is a gap in our understanding of what drives clinical injury regionally in the spine. Each of these deficits (strength, reflex-response, and proprioception) may play unequal roles in disability and also may improve differently after intervention. It is the goal of this report to describe a method for reproducibly measuring these separate neurological injuries that contribute to hand dysfunction in the CSM patient population. We additionally present how these outcomes may associate with published radiographic MRI grading systems in the literature as we hypothesize that radiographic compression will correspond with increased neuromechanical injury, associated with tract-specific injury, in this population.

## Methods

### Subjects

Thirty-seven subjects consisting of twenty adult patients and seventeen healthy controls were recruited to the study. Consent was obtained and all study related protocols were approved ahead of time by the Northwestern University IRB review board. Consent was obtained both written and oral from all study participants. All patients with clinical and radiographic signs of CSM were recruited from a single academic spine practice of five board certified neurosurgeons. Patients were included if they reported characteristic signs of cervical myelopathy, including upper extremity weakness, sensory loss, a lack of hand and leg coordination, and gait instability in combination with spinal imaging indicating cervical spine compression. All patients had a modified Japanese Orthopedic Association (mJOA) [1] myelopathy grades <18. Subjects with major neurodegenerative diseases such as Alzheimer’s or multiple sclerosis, spinal tumors or trauma, tremor, diabetes, active systemic rheumatological disease, peripheral or vascular neuropathy, and a history of spinal surgery were excluded. In addition to the exclusion criteria for patients, controls were also screened for current spinal conditions, neck pain or other neurological deficits. All participants provided written informed consent as approved by the Institutional Review Board. Demographic data including age, sex, height, and weight were assessed.

### Clinical and Health Related Quality of Life (HRQOL) assessment

Modified Japanese Orthopedic Association (mJOA) and Nurick scores were evaluated for all subjects. The mJOA score is a widely used scale to assess upper and lower extremity sensorimotor function and sphincter dysfunction, while the Nurick scale [8, 9] assesses ambulatory status. All subjects completed the neck and arm numerical rating scales (NRS), neck disability index (NDI), pain interference scale (SF-6a), and SF-36 physical (PCS) and mental component scores (MCS) (health and well-being survey).

### Neuromechanical testing

**Fig 1:**
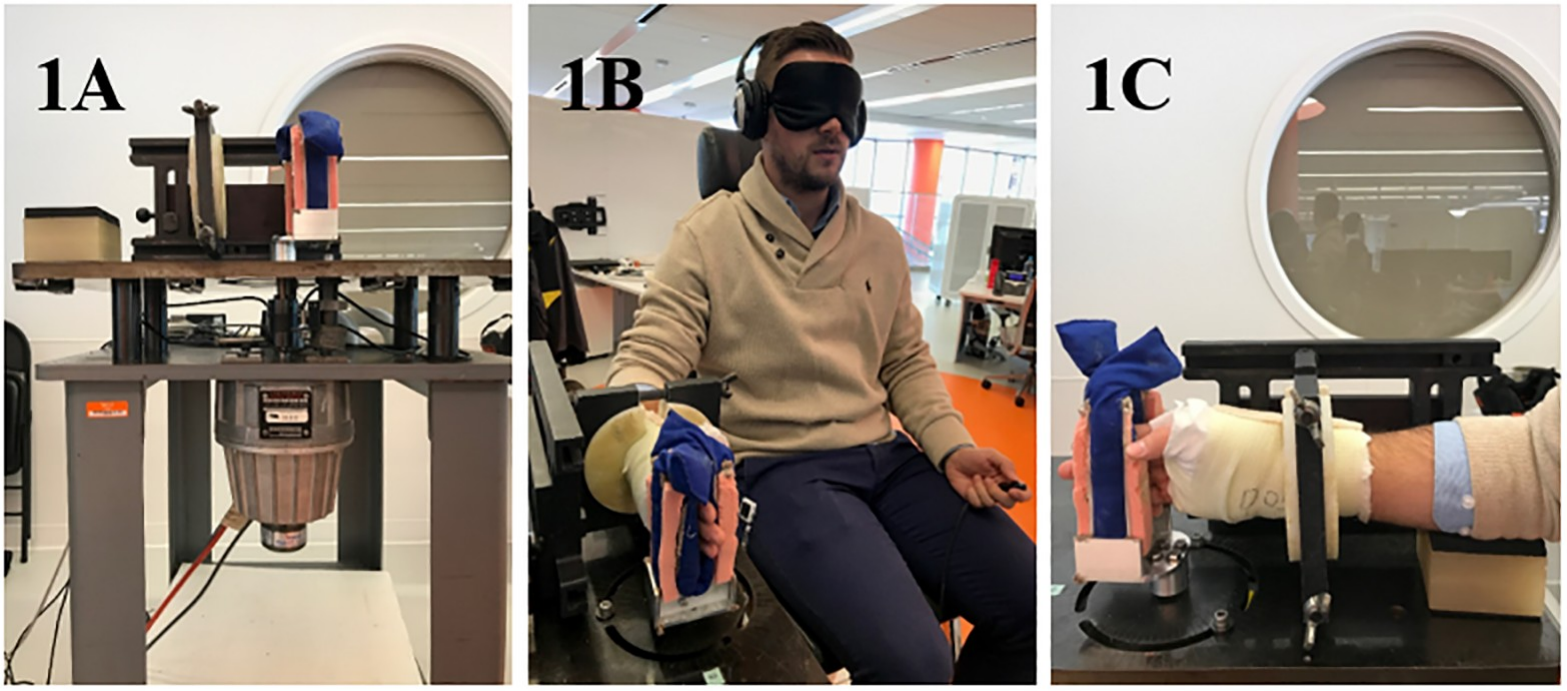
Experimental testing set-up. The set-up and motor are shown (A) as well as patient testing (B-C). Subjects are visually masked and wear noise cancellation headphones (B). The arm is temporarily casted to prevent motion proximal to the MCP joint (C).

Experimental Setup- Sensorimotor dysfunction in the hand was tested using a custom motorized apparatus adapted from Kamper et. al. as shown in Fig 1A [10]. The subject’s forearm was placed in a cast to confine motion to their metacarpophalangeal joint and maintain a neutral wrist posture with respect to the forearm (Fig 1B and 1C). The forearm was secured to the table such that their MCP joint was vertically aligned to the motor shaft, with rotation of the motor shaft producing equivalent rotational motion in the fingers. Surface Electromyographic (EMG) sensors were placed on the flexor digitorum superficialis (FDS) and the extensor digitorum communis (EDC) muscles. Angular position and velocity were recorded by the encoder (Porous Materials, Inc.; Ithaca, NY; USA) and motor tachometer (Kollmorgen; Radford, VA; USA), respectively. Range of motion in flexion and extension at the MCP joint was calculated by rotating the shaft.

Strength at the MCP joint was measured as the maximum voluntary contraction in flexion and extension directions by measurement with a torque sensor in line with the joint (Transducer Techniques; Temecula, CA; USA). Maximum Voluntary Contraction (MVC). To adjust for potential differences, this strength was normalized to age and gender [11]. All data were low-pass filtered at 200 Hz with a second-order Butterworth filter, and then sampled at 500 Hz with an analog to-digital (A/D) converter (board PCI-MIO-16E-4, National Instruments Corporation, Austin, TX; USA). Position, and velocity data were subsequently digitally low-pass filtered at 10 Hz, using a 30th-order finite impulse response filter. Raw EMG data was band-pass filtered from 20–450 Hz with hardware (Delsys Bagnoli 8-Ch EMG; Natick, Massachusetts; USA): the EMG data were then rectified, normalized and filtered with a 250 Hz, 50^th^ order low-pass filter using custom software (MATLAB). A *p-value* of < 0.05 was considered significant for statistical analysis.

### Output measures

**Fig 2:**
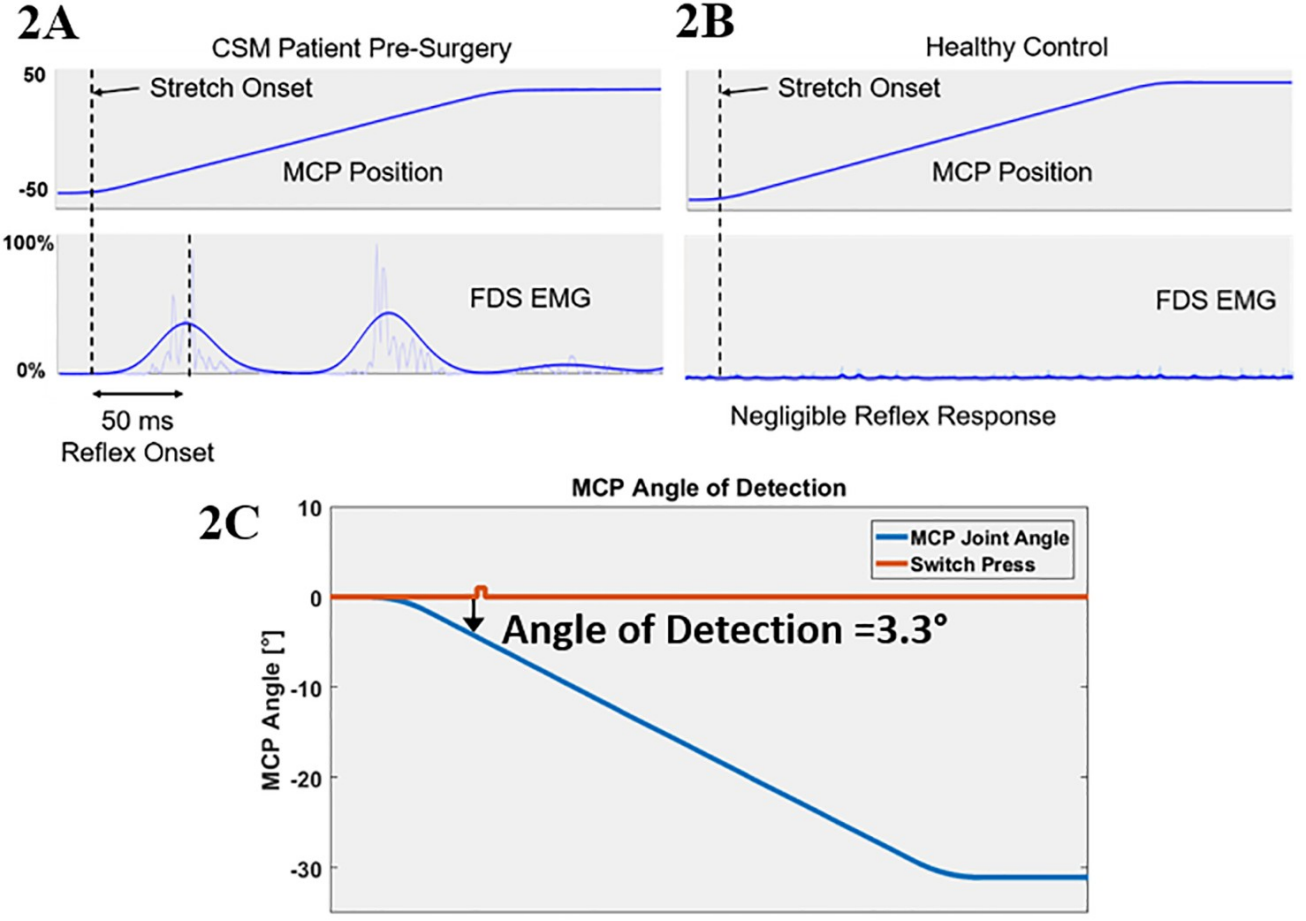
Illustration of reflex and proprioceptive testing output. FDS muscle activation and reflex during the stretch trial (A-B). Minimum angle of detection identified by switch press (C).

Hyperreflexia: Stretch trial was conducted by moving the MCP joint passively from maximum flexion to maximum extension at 300°/sec. To capture the spinal reflex response, the EMG response was taken as the peak FDS response within 50 ms of the initiation of the extension movement [12], [13]. Peak FDS EMG normalized to subject’s MVC was used as the marker for hyperreflexia. In patients FDS muscle activates as the stretch trial begins, while a control shows no major muscle activity. A graphic representation of the EMG response of a subject with CSM compared to a healthy control is demonstrated in Fig 2A and 2B.

Proprioception: Subjects were blindfolded and asked to wear noise-cancelling headphones to minimize visual and auditory proprioceptive cues. The motor passively rotated their MCP joint at 10°/sec and subjects were asked to press a button when they sensed the finger motion. The trial was repeated 10 times, out of which the motor moved five times and remained stationary for five trials. Proprioception was quantified as the minimum angle at which motion was detected by button press as shown in Fig 2C.

Strength: Subject’s strength at the MCP joint was measured as maximum voluntary contraction in flexion and extension. Strength was measured in Nm at the MCP joint by the use of a torque cell in line with the joint. An EMG output from the flexor digitorum superficialis (FDS) and extensor digitorum communis (EDC) during maximum flexion and extension, respectively, are used throughout the data analysis to normalize the EMG signal. This was utilized with maximum voluntary contraction from subject or the control. This was done so that age and gender would play less of a role in any differences between the two groups of participants.

### MRI grading scales

All imaging data were collected with a 3.0 Tesla Siemens Prisma MR scanner (Siemens, Erlangen, Germany) equipped with a 64-channel head/neck coil. Axial and T2 weighted sequences were acquired. Participants were placed supine on the scanner bed, and a localizer scan was obtained to identify the location of the intervertebral discs of the cervical spine (C2–3, C3–4, C4–5, C5–6, C6–7, and C7-T1). Several published grading scales from the literature were identified and calculated, namely Nagata et. al.[14], Kang et. al. [15], Chang et. al. [16], modified Torg-Pavlov ratio (defined as the ratio of the anterior-posterior measurements of the spinal canal and the vertebral body at the level of worst compression, as measured in the sagittal plane) [17]. Compression ratio (defined as the ratio of the anterior-posterior and transverse cord dimensions) and cross-sectional area at the level of compression were calculated. These scales assign a numerical value to the stenosis depending upon the severity of spinal cord compression, presence of cerebrospinal fluid (CSF) around the cord, signal change, axial cord distortion and cord occupancy in the canal. A board-certified radiologist, who was not associated with the patient’s clinic care, assessed the compression grades at each level of the cervical spine. The radiologist was blinded to patient symptomatology during interpretation of the MR imaging. Maximum compression score from each grading system was used for further analysis.

### Statistical analysis

Normality of the data was tested using the Shapiro-Wilk test. Log transformation were applied to normalize the data, and parametric tests- 2 sample t-test, linear regression multivariate modeling was used to determine mean differences and relation between demographic data, clinical scores, and neuromechanical parameters among patients and controls. Spearman’s correlation was conducted to assess relation between compression on MRI and clinical/HRQOL scores.

## Results

### Subject characteristics- demographic, radiographic, and clinical & HRQOL scores

The patient cohort consisted of 20 subjects (eight females and twelve males) while the control group consisted of 17 subjects (8 females and 9 males); mean ages 58.8 ± 12.2 years and 52.8 ± 9.3 years respectively (p = 0.089). Patients had significantly greater compression score, and worse clinical and HRQOL scores. Patients had a higher Nurick (1.90±1.0 vs 0±0, p<0.001) and lower mJOA scores (14.3±1.9 vs 18.0±0, p<0.001) as compared to controls. Patients with CSM reported higher neck discomfort (4.0±3.2 vs 0.2±0.4, p<0.001), arm discomfort (4.5±2.6 vs 0.35±0.70, p<0.001), NDI (16.4±10.6 vs 1.47±2.5, p<0.001) and SF-6a (58.51 ±9.6 vs 42.2±4.5, p<0.001); and lower PCS (41.8 ±10.7 vs 57.1 ±4.2 p<0.001) and MCS (47.6 ±9.6 vs 57.1±4.2, p<0.001) scores. Patients also showed significantly higher spinal compression indicated by Nagata (χ^2^(3) = 18.53, p<0.001), Chang (χ^2^(2) = 14.22, p<0.001), Kang (χ^2^(4) = 12.4, p = 0.015), compression ratio (0.396±0.07 vs 0.53±0.08 p<0.001), and m-Torg-Pavlov ratio (0.58±2.0 vs 0.29±0.6 p<0.001); and lower cross-sectional area (67.6±11.9 vs 80.7±9.6, p = 0.004) as compared to controls. The patient’s demographic data, clinical and HRQOL scores are listed in Table 1.

**Table 1:** Controls and CSM patients’ demographic data, including age, gender, clinical myelopathy grades, and HRQOL scores; Mean±S.D. are reported.

	Subject	M/F	Age	Nurick	mJOA	Arm NRS	Neck NRS	NDI	PCS	MCS	SF-6 a	Max Level of Compression
Controls (n = 17)	C1	M	51	0	18	0	0	0	58.47	53.61	41.1	C5-C6
	C2	M	53	0	18	0	0	0	57.93	57.59	41.1	C3-C4
	C3	F	55	0	18	0	0	0	60.81	57.55	41.1	C4-C5
	C4	F	55	0	18	0	1	0	55.84	52.92	41.1	C5-C6
	C5	F	55	0	18	0	0	1	58.72	62.26	41.1	NA
	C6	M	67	0	18	0	0	0	55.91	60.68	41.1	C5-C6
	C7	M	51	0	18	0	1	0	56.67	58.2	41.1	C5-C6
	C8	F	63	0	18	2	1	9	44	49.99	59.5	C4-C5
	C9	M	54	0	18	0	0	1	60.53	58.27	41.1	C4-C5
	C10	F	42	0	18	0	0	0	56.72	60.24	41.1	C6-C7
	C11	M	45	0	18	0	0	0	58.15	56.98	41.1	C5-C6
	C12	M	26	0	18	2	0	4	57.98	55.58	41.1	C5-C6
	C13	M	62	0	18	1	1	4	51.24	58.23	41.1	C6-C7
	C14	F	61	0	18	0	0	0	56.3	57.63	41.1	C5-C6
	C15	M	50	0	18	1	0	2	61.2	52.9	41.1	C5-C6
	C16	F	50	0	18	0	0	4	60.7	56.9	41.1	C4-C5
	C17	F	52	0	18	0	0	0	59.5	57.5	41.1	C3-C4
	**Mean**	**9M/8F**	**52.47**	**0**	**18**	**0.35**	**0.24**	**1.47**	**57.1**	**56.88**	**42.18**	
	**SD**		**9.32**	**0**	**0**	**0.7**	**0.44**	**2.48**	**4.17**	**3.1**	**4.46**	
Patients (n = 20)	P1	F	62	1	15	3	0	10	53.34	45.3	52.2	C3-C4
	P2	M	70	2	12	6	5	12	46.2	48.09	62.1	C5-C6
	P3	M	67	3	11	9	9	39	28.79	35.53	71	NA
	P4	M	54	2	12	5	7	25	42.01	63.21	61.2	C6-C7
	P5	F	60	4	13	5	4	10	31.26	60.57	52.2	C3-C4
	P6	M	60	1	17	3	4	8	52.16	52	52.2	C5-C6
	P7	M	72	2	13	9	8	31	48.44	28.06	76.3	C4-C5
	P8	F	50	1	16	1	1	14	57.29	39.12	56.6	C3-C4
	P9	M	63	1	15	4	3	17	43.31	47.67	55.6	C5-C6
	P10	M	68	2	12	1	4	4	51.62	57.35	48.6	C5-C6
	P11	M	64	2	14	3	4	24	26.42	36.95	66.7	C5-C6
	P12	M	60	3	14	7	9	24	25.76	43.44	69.8	C6-C7
	P13	F	66	2	15	4	6	14	41.73	39.91	63	C5-C6
	P14	F	58	1	17	0	0	0	55.61	54.89	41.1	NA
	P15	F	61	2	13	6	8	22	34.57	43.95	63.8	C5-C6
	P16	M	36	2	17	4	0	5	45	57.8	50.7	C6-C7
	P17	M	25	1	13	5	2	11	44.5	56	54.5	C5-C6
	P18	F	63	1	16	8	0	20	38.2	44.4	64.8	NA
	P19	M	43	1	17	1	0	5	49	54.2	41.1	C5-C6
	P20	F	74	4	14	6	6	33	21.2	44.6	66.7	C5-C6
	**Mean**	**12M/8F**	**58.8**	**1.9**	**14.3**	**4.5**	**4**	**16.4**	**41.82**	**47.65**	**58.51**	
	**SD**		**12.2**	**0.97**	**1.92**	**2.63**	**3.2**	**10.58**	**10.68**	**9.25**	**9.61**	

mJOA = modified Japanese Orthopedic Association, NRS = Numerical rating system, NDI = Neck Disability Index, PCS = Physical Component Score, MCS = Mental Component Score, SF6a = Pain Interference.

### Neuromechanical deficits and clinical scores

**Fig 3:**
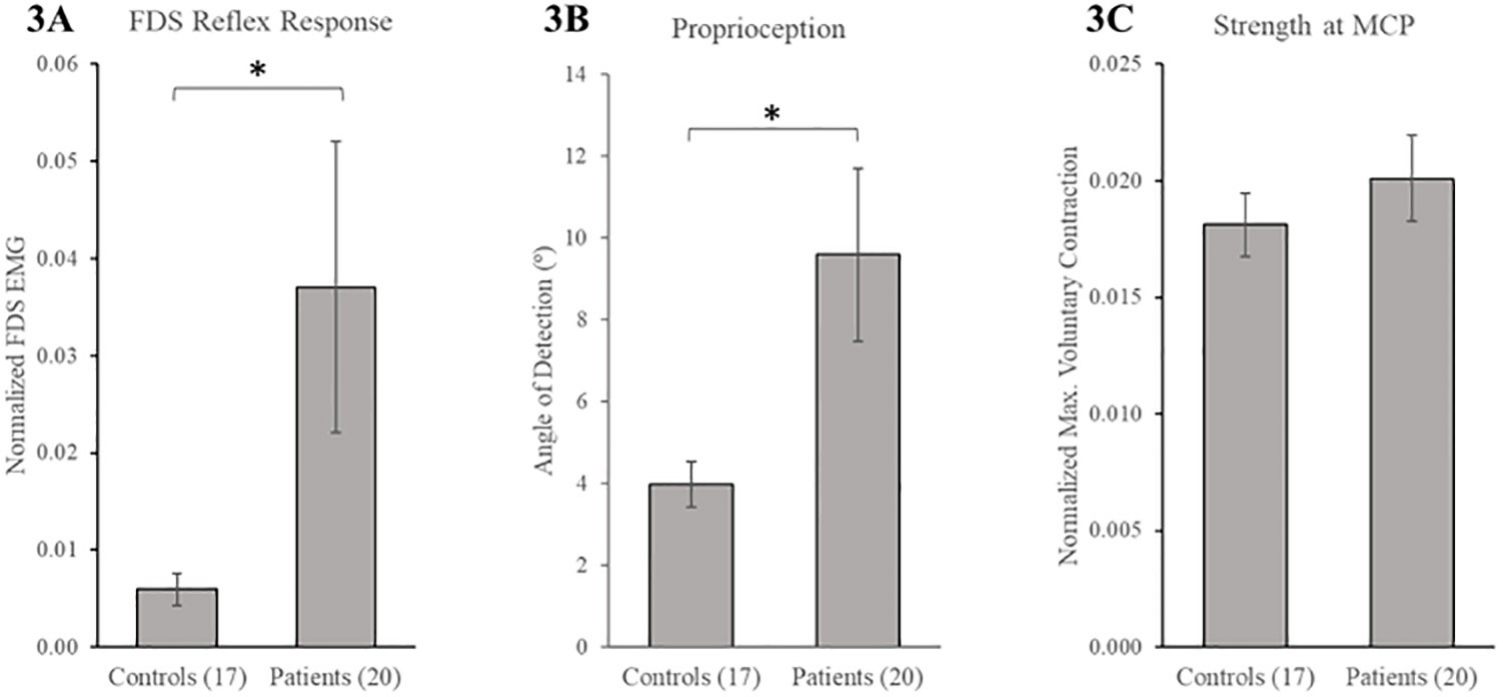
Neuromechanical measures between patients with CSM and controls. Hyperreflexia as measured by normalized peak FDS EMG (A). Proprioception as measured by minimum angle of detection (B). Strength measured as normalized MVC (C). Mean (SE) are reported. *Denotes significant differences (p<0.05).

Neuromechanical measures quantifying sensorimotor deficits differed significantly between CSM and healthy controls. Specifically, patients with CSM had increased reflexes (normalized peak FDS EMG) (0.0370±0.067 vs. 0.006±0.007, p = 0.007) (Fig 3). Patient proprioception was significantly worse; mean angle of detection was greater than twice as high in patients (9.6± 9.43°) compared to controls (4.0± 2.3°), p = 0.022. The mean strength at the MCP joint (normalized MVC) in patients was 0.020±0.008, while in controls the mean was 0.018±0.005. There were no significant differences in MCP strength between patients and controls (p = 0.40). Similarly, neuromechanical measures were also found to be associated with commonly used clinical scores in myelopathy. A linear regression multivariate model of mJOA showed that normalized peak FDS EMG (*β* = -1.060, p = 0.001) was the most significant predictor of sensorimotor deficits. The model explained 42.1% of variance (R^2^ = 0.421); *F*(1, 35) = 24.493, p = 0.001. When compression ratio was added to the multivariate model, it was also a significant predictor of mJOA (*β* = 8.765, p = 0.029), along with angle of detection (*β* = -0.158, p = 0.006) and FDS EMG (*β* = -0.526, p = 0.039). It improved the overall accuracy of the model (R^2^ = 0.578); *F*(3, 28) = 12.807, p = 0.001.

### Diagnostic accuracy of neuromechanical parameters

Hyperreflexia and declining proprioception were fairly accurate (area under the curve = 85.0% and 67.1% respectively) in identifying patients with CSM from controls. At a threshold of 0.0066, peak FDS EMG identified 85.0% of patients and 76.5% controls correctly. Angle of detection of 2.6° was 90% sensitive and 29.4% specific in classifying patients from controls. When the cut-off was increased to 8.5°, angle of detection correctly identified 94.1% controls but only 40% of patients.

### Neuromechanical parameters and HRQOL scores

Neuromechanical measures of hyperreflexia and proprioception were also significantly associated with patient reported HRQOL measures. Increased reflexes (normalized peak FDS EMG predicted higher neck disability (NDI; r = 0.610, p = 0.001) and lower physical function scores (SF-PCS; r = -0.696, p = 0.001, Neck NRS; r = 0.608, p = 0.001, Arm NRS; r = 0.637, p = 0.001, SF6a-Pain; r = 0.707, p = 0.001). Worse proprioception (angle of detection) was associated with higher neck (NRS; r = -0.371, p = 0.024) and arm (NRS; r = -0.361, p = 0.028) discomfort, and physical function scores (SF-PCS; r = -0.354, p = 0.032).

### MRI compression scales and neuromechanical deficits

**Fig 4:**
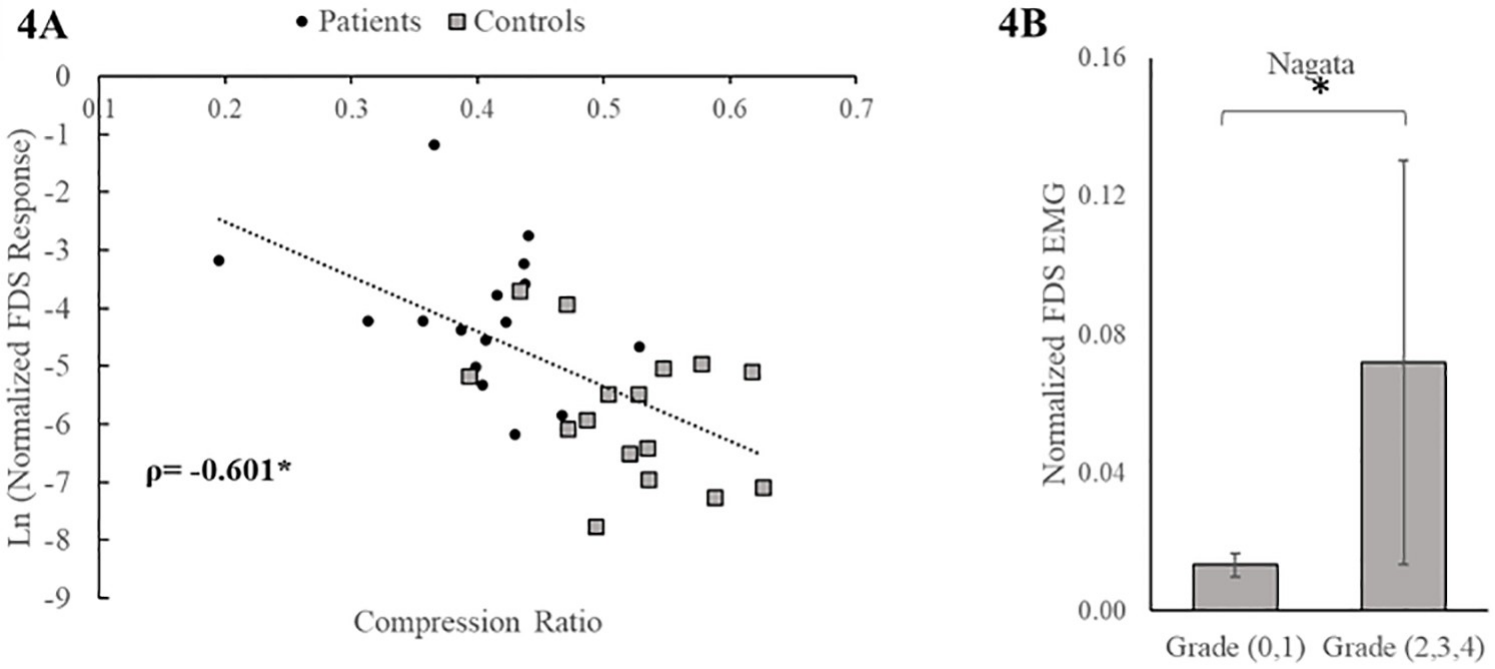
Association between hyperreflexia and MRI based compression grades. Increased reflexes (peak FDS EMG) were associated with higher spinal cord compression measured by compression ratio (A) and Nagata grading system (B). *Denotes significance at p<0.05.

MRI compression grades considered in this study- Nagata, Kang, Chang, compression ratio, m-Torg-Pavlov and cross-sectional area showed strong, significant agreement in classifying spinal compression severity among each other. Higher compression on these MRI scales predicted significantly higher impairment on clinical scales- mJOA and Nurick. Higher compression scores on MRI grading scales were also significantly associated with decreased patient reported HRQOL indices of neck disability, pain interference and physical function (Table 2). Several MRI compression grading scales, namely Nagata, compression ratio and m-Torg-Pavlov ratio were significantly related to neuromechanical measures. Compression ratio significantly predicted increased reflexes in patients (normalized first peak FDS EMG; ρ = -0.601, p = 0.001). Log transformed FDS EMG values show that a decline in compression ratio resulted in an increase in reflexes (Fig 4A). However, it was not associated with proprioceptive angle (ρ = 0.012, p = 0.949) or strength at MCP joint (ρ = -0.120, p = 0.695). Modified Torg-Pavlov ratio showed significant association with reflexes (ρ = -0.659, p = 0.001). In the Nagata scale, upon dichotomous sub classification based on presence of compression of spinal cord (grade 0, 1 vs 2, 3, and 4), reflexes were significantly higher among the higher-grade scores (0.034±0.071 vs. 0.007±0.011, p = 0.007) (Fig 4B) but proprioception (7.29±6.7 vs. 4.14±2.40, p = 0.087) and strength (0.022±0.008 vs. 0.018±0.005, p = 0.131) were not statistically different. Cross-sectional area at the level of compression correlated negatively with increased reflexes but did not reach significance (ρ = -0.255, p = 0.219). Kang and Chang scales performed identically when classifying subjects into severe vs mild/moderate compression grades. Strength (normalized MVC) was significantly different between the two groups; however, Chang and Kang scales were not sensitive to changes in hyperreflexia or proprioception.

**Table 2:** Correlation analysis between MRI grading systems, clinical scores and HRQOL life scores. Spearman’s rho and corresponding p values are reported.

**Rho**	**Nagata**	**Kang**	**Chang**	**Comp ratio**	**m- Torg Pavlov**	**CS Area**	**mJOA**	**Nurick**	**Neck NRS**	**Arm NRS**	**NDI**	**SF-PCS**	**SF-MCS**	**SF6a Pain**
**(p value)**														
**Nagata**	1.000	0.818	0.809	-0.746	-0.841	-0.691	-0.660	0.735	0.573	0.392	0.641	-0.612	-0.353	0.629
		0.000	0.000	0.000	0.000	0.000	0.000	0.000	0.001	0.026	0.000	0.000	0.047	0.000
**Kang**	-	1.000	0.800	-0.551	-0.680	-0.452	-0.510	0.571	0.429	0.270	0.473	-0.506	-0.237	0.501
			0.000	0.001	0.000	0.023	-0.002	0.001	0.014	0.135	0.006	0.003	0.191	0.003
**Chang**	-	-	1.000	-0.679	-0.857	-0.566	-0.670	0.780	0.582	0.481	0.576	-0.703	-0.225	0.659
				0.000	0.000	0.003	0.000	0.000	0.000	0.005	0.001	0.000	0.215	0.000
**Comp ratio**	-	-	-	1.000	0.712	0.428	0.674	-0.713	-0.628	-0.467	-0.592	0.659	0.357	-0.665
					0.000	0.033	0.000	0.000	0.000	0.007	0.000	0.000	0.045	0.000
**m -Torg Pavlov**	-	-	-	-	1.000	0.634	0.724	-0.807	-0.643	-0.600	-0.590	0.681	0.265	-0.683
						0.001	0.000	0.000	0.000	0.000	0.000	0.000	0.143	0.000
**CS Area**	-	-	-	-	-	1.000	0.375	-0.453	-0.244	-0.324	-0.379	0.408	0.265	-0.358
							0.064	0.023	0.241	0.114	0.062	0.043	0.201	0.079

### Repeated testing following intervention

**Fig 5:**
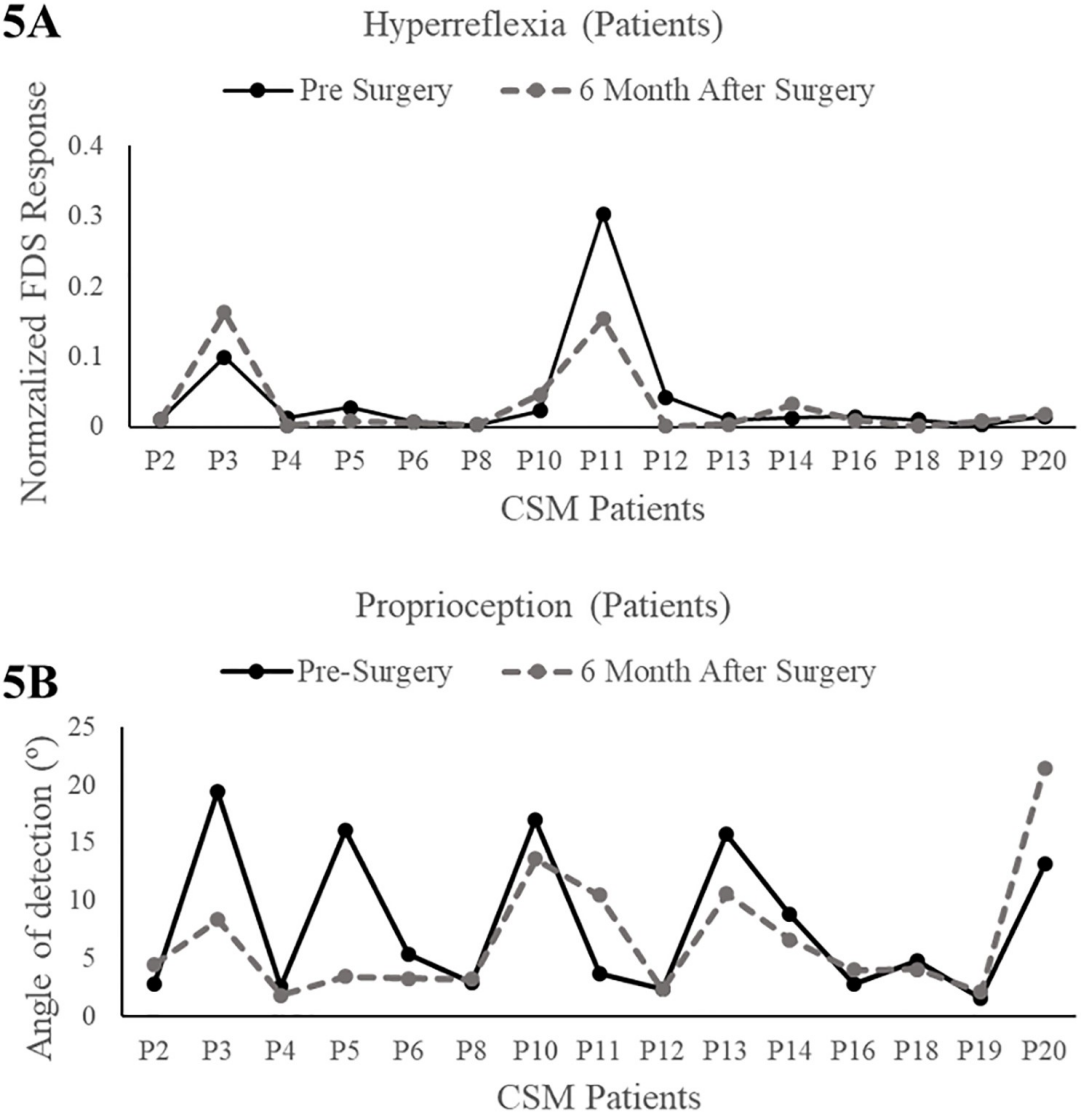
Preliminary findings. Improvement in neuromechanical function in patients after surgical intervention. Reduction in reflexes (A) and proprioceptive angle of detection (B).

We conducted repeated testing in both patients, following surgical intervention, as well as control subjects. The follow-up data in 13 controls and 15 patients who have reached 6 months following intervention (or time to repeat testing in controls) are plotted in Fig 5A and 5B. Our preliminary results show that patients with CSM experience improvements in neuromechanical function of the hand. The proprioceptive marker—minimum angle of detection reduces by ~3° from 9.5 to 6.6° in patients on an average. Most patients show reduction in hyperreflexia within first 6 months of their recovery however, a few patients show increase in their FDS EMG response.

### Discussion

Cervical spondylotic myelopathy is a potentially debilitating condition with decompressive surgery the cornerstone of treatment [2, 3, 18]. A patient’s clinical disability is likely the aggregate of multiple ascending and descending pathway deficits. The goal of the current study was to develop objective and repeatable means of testing isolated neurological deficits in patients with this disease, including the loss of strength, diminished proprioceptive skill, and increased reflex response. To our knowledge, no previous patient series with quantified measures of strength, proprioception, and reflex response have been reported in this patient population. The goal of this work is to prove that these deficits can be isolated consistently, a critical step in better understanding the neurological drivers of injury in this patient population.

Cervical myelopathy may strongly impact both the upper and lower extremities. Clinical disability, however, is heavily driven by hand dysfunction. Injury to the corticospinal pathways may result in both motor weakness as well as finger spasticity. Characteristic findings include an inability to adduct and extend the fingers as well as grip and quickly release objects in the hand [19]. Examinations in cervical myelopathy patients also will commonly reveal joint proprioception and two-point discrimination deficits [20, 21]. Thus, injury to the dorsal columns likely is also a critical component of hand dysfunction. Doita et al. reported a direct relationship between clinical myelopathy and proprioceptive ability in a hand function test [22]. In a study comparing CSM patients and normal controls, Akutagawa et al., found that myelopathy patients required a higher pinch pressure to grasp and hold small objects [23]. These studies suggest that impairment of the hand may be related to injuries to both the motor and sensory spinal tracts of the cervical spinal cord. Consistent with these studies, neuromechanical metrics used in this study have characterized patients with cervical myelopathy and their associated motor and sensory deficits, as indicated by increased reflexes and reduced proprioception respectively.

In the current study, we examined implied pyramidal tract impairment as manifested by both loss of strength and finger hyperreflexia. However, the present results did not indicate any significant difference in age and gender matched strength between cervical myelopathy patients and controls. In contrast, there was a significant difference between recorded levels of finger hyperreflexia (reflex response) when myelopathy patients were compared to controls. In the multiple regression model, hyperreflexia was additionally a significant predictor of both mJOA and Nurick score. These findings are in agreement with reported clinical observations. Houten and Noce reported that in patients with severe myelopathy, a Hoffman sign for finger hyperreflexia was found in 81% of patients [24]. Nemani et. al. reported that increased pre-operative reflexes as well as the presence of pathologic reflexes were strongly predictive of imaging characteristics of spinal cord compression and pathologic spinal cord signal change [25]. Our current findings suggest that quantifiable hyperreflexia may be a sensitive measure for identifying patients with clinical myelopathy.

To investigate proprioceptive deficits, we evaluated the angle to detection for CSM patients and controls. We report that the angle for detected movement increases significantly for CSM patients when compared to controls. Proprioception was additionally a predictor of clinical myelopathy (mJOA and Nurick) using the multiple regression analysis. These results may reflect dorsal column dysfunction in this patient cohort. Many tests have been developed to evaluate the function of the hand, including proprioceptive skill. However, there is no consensus on the best testing protocol. The current study’s neuromechanical metrics were extracted independent of functional tasks, at an isolated joint, and can be reliably repeated from examiner to examiner.

Recent studies have shown that chronic injury to the cervical spinal cord with spondylosis can be non-uniform and associated with tract-specific injury [26]. We have previously shown that anterior spinal cord compression and injury, as quantified by advanced MRI metrics, will predict symptoms of clinical myelopathy [27]. Further, our work shows that that specific descending motor tracts (ex: reticulospinal tract) may be most heavily impacted by cervical spondylosis [28]. In the current work, we utilized previously published cervical myelopathy grading scales to determine a grade of compression for each clinical subject. We additionally utilized a MRI modification of the Torg-Pavlov ratio [6, 17], patterned off the established, pre-MRI era, Torg-Pavlov ratio for canal capacity, alongside several published MRI scales used to predict CSM by imaging characteristics alone.[6, 14, 15, 16, 25, 29] We found that while most MRI grades had concurrent validity and were related to clinical scores and HRQOL scores, only a couple, namely Nagata grades and compression ratio, were predictive of testing for cervical myelopathy. This suggests that the neuromechanical measures utilized in this study are reflective of anomalous spinal cord characteristics such as increased spinal cord distortion and loss of cerebrospinal fluid around the cord. This further underscores the need to define objective measures of spinal cord compression that are better associated with sensorimotor dysfunction and predictive of CSM.

We acknowledge there are a number of limitations in the current study. First, although we sought to study a range of patients with cervical myelopathy, the majority of the study patients had either mild or moderate symptoms. Second, we acknowledge that the current device and use of temporary arm immobilization would not be currently feasible in a busy clinical practice. A less cumbersome device and set-up would be needed. In this work, our primary goal was to develop a means to isolate specific deficits in this population. Third, the CSM patient often will present with a complex array of symptoms, including neck pain, upper extremity dysfunction, and lower extremity deficits. While the current work focuses on deficits in the hand, gait disturbance and lower extremity motor impairment are often critical features of symptomatic cervical myelopathy. Although beyond the scope of the current work, any comprehensive neuromechanical evaluation of the CSM patient will require testing of the lower extremity, including both tests of power and gait.

We believe the diagnosis of cervical myelopathy is always a cumulative picture of a patient’s history, neurological imaging, and clinical exam findings. As our field evolves, both refinements in imaging and clinical assessments may improve our ability to detect signs of spinal injury in CSM patients with improved precision and sensitivity. Although the neuromechanical testing variables presented here may not outperform new advances in MRI imaging (ex: DTI-MRI), we do believe neurological testing is a critical part of developing a model for decision making that includes thresholds for intervention and continued follow-up. We believe this would enhance, not replace, the judgment of clinician and surgeon.

This study describes a translational testing system to quantify individual neurological deficits that contribute to hand dysfunction in CSM patients. This quantitative assessment tool provides evidence that separate tract-specific deficits can be reproducibly isolated in CSM patients and that increased reflex response and proprioceptive deficits are likely the primary drivers of hand dysfunction. These neurological changes can be tested both before and after surgical intervention and found to improve with surgical intervention [30]. Further, while there remains no single consensus MRI grading scale, radiographic compression does associate with neurological deficit. The most sensitive measure appears to be eccentric distortion of the spinal cord. Given the current reliance on subjective and non-quantitative measurements, the current technique can inform the design of clinically feasible devices, guide evaluations of emerging MRI technologies, and improve our understanding of the drivers of neurological injury in this population.

## Supporting information

S1 File Demographic, clinical and health related quality of life scores, MRI based compression grades and neuromechanical data for all the subjects. (CSV)

## Abbreviations

AUC: Area Under Curve
BMI: Body Mass Index
CSF: Cerebrospinal Fluid
CSM: Cervical spondylotic myelopathy
EDS: Extensor Digitorum Communis
EMG: Electromyographic
FDS: Flexor Digitorum Superficialis
HRQOL: Health Related Quality of Life
MCP: Metacarpophalangeal
MCS: Mental Component Score
mJOA: modified Japanese Orthopedic Association
MRI: Magnetic Resonance Imaging
MVC: Maximum voluntary contraction
NDI: Neck Disability Index
NRS: Numerical Rating Scales
PCS: Physical Component Score
